# Patients with heart failure as co-designers of an educational website: implications for medical education

**DOI:** 10.5116/ijme.5898.309e

**Published:** 2017-02-25

**Authors:** Anne Mette Kristiansen, Jette R. Svanholm, Inge Schjødt, Karsten Mølgaard Jensen, Charlotte Silén, Klas Karlgren

**Affiliations:** 1 Department of Cardiology, Aarhus University Hospital, Denmark; 2 Department of Learning, Informatics, Management and Ethics, Karolinska Institutet, Sweden

**Keywords:** Patient education, heart failure, learning needs, website, mixed methods approach

## Abstract

**Objectives:**

To identify the learning needs of patients with heart failure between outpatients follow-up visits from their perspective and to ascertain what they emphasize as being important in the design of an educational website for them.

**Methods:**

We conducted a two-step qualitative study at Aarhus University Hospital, Denmark. Twenty patients with heart failure participated either in focus group interviews, diary writing, or video-recorded design sessions. Data on learning needs were collected in step 1 and analyses, therefore, helped develop the preliminary prototypes of a website. In step 2, patients worked on the prototypes in video-recorded design sessions, employing a think-aloud method. The interviews were transcribed and a content analysis was performed on the text and video data.

**Results:**

Patients’ learning needs were multifaceted, driven by anxiety, arising from, and often influenced by, such daily situations and contexts as the medical condition, medication, challenges in daily life, and where to get support and how to manage their self-care. They emphasized different ways of adapting the design to the patient group to enable interaction with peers and professionals and specific interface issues.

**Conclusions:**

This study provided insights into the different learning needs of patients with heart failure, how managing daily situations is the starting point for these needs and how emotions play a part in patients’ learning. Moreover, it showed how patient co-designers proved to be useful for understanding how to design a website that supports patients’ learning: insights, which may become important in designing online learning tools for patients.

## Introduction

Successfully performed self-care in the context of heart failure is a complex matter.  Heart failure is a serious chronic cardiac condition^1^ in which the heart’s capacity to transport enough blood is weakened. This can result in, e.g., such signs as fluid retention and such symptoms as dyspnea, fatigue, etc.,^2^ and requires lifelong and constant patient involvement to carry out adequate, continuous self-care. Unfortunately, many patients with heart failure fail to achieve adequate self-care behavior, such as following medical prescriptions, which leads to readmissions.^3^  

There is a need to gain more knowledge about how to support these patients. In this study, the patients’ perspective on their needs will be the basis for the development of a website supporting self-care. It is a well-known fact that patients’ self-care behaviors need to be improved to reduce mortality, morbidity, and healthcare costs.^4^  This becomes ever more important since the annual incidence of heart failure is increasing.^1^^,^^5^^-^^7^ Teaching self-care behaviors is already a key element in patient education and plays a vital part in heart failure management programs^3^  aiming at supporting patients in the learning and development of various competencies.^8^ However, these activities do not seem to adequately support patients’ learning concerning how to deal with a chronic cardiac condition in everyday life. It is also to be expected that the majority of patients with heart failure might need to update and expand their knowledge and skills in this area over time in order to be able to handle more and more information. The reason for this is that their condition and how it is managed over time can vary. Being diagnosed with heart failure might require the patient to make changes and learning is a prerequisite for such change.^9^

Having a chronic disease involves spending most of one’s time away from the hospital;^10^ thus, learning occurs primarily in everyday life and everyday contexts. An educational website could, therefore, be a suitable tool to support patients living with heart failure. Studies^3^^,^^11^   have suggested that computer-assisted learning can address some of the challenges posed by heart failure and the attempts at using computer-assisted learning have proved to be efficient in increasing patients’ knowledge and skills. Computer-assisted learning has the potential to address needs, and wishes of the patients^12^^,^^13^ and some^3^^,^^14^  have argued for bringing patients’ views into the process of developing computer-assisted learning tools. However, only a few studies have addressed this within the area of heart failure.^15^^,^^16^ 

In general, research on patient education concerning heart failure tends to focus on such topics as general information about the condition, monitoring of symptoms and self-care, pharmacological and non-pharmacological management, rest and exercise, fluid restriction, etc.^1^^,^^3^^,^^17^  Although previous research^18^^-^^25^ has discussed the learning needs of patients with heart failure; there is a lack of knowledge about what patients consider to be important to learn. This lack of knowledge about the patient perspective undermines the quality of patient education. Concern for the quality of patient education and patient learning is a professional responsibility and, in order to understand how we can offer better patient education and support patients’ learning and management of heart failure, we need to take the patients’ perspective into account. The present study was based on the assumption that patients need to learn to be able to cope with their condition. Thus, the findings will be interpreted in relation to learning theories.

To sum up, patients with heart failure often fail to establish crucial self-care behaviors and there is a need to learn more about how they can be supported. They spend much time away from the hospital and need to learn more about their condition over time. While digital support between visits to hospitals may address these needs, there is a lack of research on the patients’ perspective on learning about heart failure. The aims of this study were, first, to identify the learning needs of patients with heart failure between outpatient follow-up visits to an outpatient heart failure clinic and, second, to ascertain what patients emphasized as being important in the design of an educational website intended to support their learning.

## Methods

### 

A qualitative research approach was chosen and we also chose to use several sources of data. We used an inductive content analysis to scrutinize data.^26^ The study was carried out within a constructivist research tradition as the aims were to understand the patients’ meaning-making and gain insight into their opinions and perceptions.^27^^, ^^28^

### Participants

Specialist nurses from the heart failure clinic identified potential participants. Inclusion criteria were: 18 years of age or older, ability to understand Danish and treatment at the heart failure clinic. Twenty-four patients with heart failure, males (n = 18) and females (n = 6), were sampled purposefully and invited to participate either in focus group interviews, diary writing, or design sessions. This approach to sampling allowed us to include patients who could provide new information in the field.^27^^,^^29^   As described by Jaspers,^30^  patients who would be potential users of the website were selected to participate in the design sessions. Twenty patients (15 males and five females) agreed to participate in the study. Characteristics of the included participants are shown in Table 1.

**Table 1:** Characteristics of the participants included in the three groups

Participants	Focus group interviews (n = 10)	Diary writing (n = 6)	Design sessions (n = 4)
Females (n)	2	2	1
Males (n)	8	4	3
Years of age (range)	47–78	48–71	65–70
Months with the heart failure diagnosis (range)	1–12 months	2–12 months	1–3 months

This study was approved by the head of the Department of Cardiology at Aarhus University Hospital and has been carried out in accordance with the Declaration of Helsinki. It was approved by the Danish Data Protection Agency and required no ethical approval according to the Central Denmark Region Committees on Biomedical and Research Ethics. Participants with advanced heart failure were not included because this could have been too burdensome for them. All participants received both oral and written information before inclusion. They were informed that their involvement was voluntary, that they could withdraw from the study without any consequences at any time and that their answers would be anonymized. All participants gave their written informed consent.

### Data collection

This study was conducted in two steps using different methods. In *Step* 1, patients described their learning needs in focus group interviews or diaries. In *Step* 2, patients participated in video-recorded design sessions employing a think-aloud method. Findings from Step 1 were used in Step 2. This means that the learning needs discovered in the analysis of data from focus group interviews and diaries were used as a basis for developing prototypes of the website.

#### Step 1 - Use of focus group interviews and diaries to investigate learning needs

Initially, we were interested in how patients perceived their own learning needs regarding heart failure during the time between follow-up visits.

Focus group interviews can give insight into the patients’ personal stories, feelings, etc.^31^^-^^34^ We developed an interview guide and pilot-tested it with two patients on separate occasions. This led to changes in wordings and expansion of one question. This interview guide was a driving force in the interviews.^28^^,^^35^  In mid-February, 2015, two focus group interviews were conducted by the first author (AMK) and one observer (JRS/IS). Both interviews lasted 60 minutes. The participants’ understanding of their own learning needs, questions and concerns were probed in depth during the interviews.

During the same period, six participants kept a semi-structured diary for two weeks. Patients were asked to focus on their daily life and especially any concerns dealing with heart failure. Questions to prompt reflection when writing were, for instance: “*What situations have you encountered today where you lacked knowledge or had questions about your heart condition*?” “*What would be important for you to learn*?” Diary writing was chosen based on the argument that patients’ learning needs do not arise solely when patients visit the heart failure clinic. Diaries can provide detailed descriptions of a patient’s everyday life and capture thoughts and feelings in challenging situations.^36^  Diaries are also likely to provide a more exhaustive picture of a patient’s activities^37^  and enable better management of memory recall problems since the data are collected closer to the real-life episodes.^38^   Diary writing can help to expose practices and habits which would otherwise risk being forgotten and allow a subject to be investigated over time and different contexts, all of which was highly relevant in this study. Verbal and written information on how to use the diaries was provided. The patients returned the diaries in a prepaid envelope or by e-mail.

#### Step 2 – design of the website prototypes

In mid-March, 2015, the participants took part as co-designers of four video-recorded design sessions, each lasting 30–45 minutes. Co-design was used in a broad sense in which a designer, the researcher, and the patients worked on the design together.^39^ Design sessions were carried out with one patient at a time. An organizer of the design process (AMK) acted as facilitator, together with the system engineer (KMJ) responsible for web design. The intention underlying these design sessions was to allow the participants to interact with prototypes of the website and to provide space for the expression of thoughts about the website design and content. These immediate thoughts about the design were triggered by open-ended questions, such as “*if you think about the potentials of a website, can you think about how these topics and subtopics might be used on a website?*” In addition, the participants were presented with certain tasks and asked to think aloud while solving them using the prototypes. The participants were encouraged to express ideas and thoughts related to the further design. Data also included written notes collected in a semi-structured protocol by the facilitator and project representative; these data were supplementary.

### Setting

This study was conducted at the Department of Cardiology at Aarhus University Hospital in Denmark. The department is highly specialized in diagnostics and state-of-the-art management of all aspects of heart disease and is responsible for the clinical follow-up of patients with heart failure in the local uptake area. This study was conducted between follow-up visits and refers to the time patients spent at home between visits to the outpatient heart failure clinic.

### Data analysis

Qualitative content analysis was used to analyze data. The focus was either on the manifest (what the text actually says) or the latent (underlying meaning) content of the data^26^^,^^40^^,^^41^ and all authors collaborated on different parts of the analysis. The focus group interviews were recorded and transcribed by the first author (AMK). The unit of analysis was the complete interview transcript. Ambiguities in diary texts were clarified by the diary keeper; the text from each diary was seen as one unit of analysis. All units of text were read and re-read several times to get a sense of the whole context. Subsequently, an inductive content analysis was carried out. First, meaning units were recognized, condensed, and abstracted into categories, i.e., what was said in the focus group interviews or written in the diaries (manifest content). Next, the latent content of the categories were formulated into themes (Table 2). The findings were then triangulated. Two members (JRS/IS) of the research team carried out 1/3 of the analysis of either the focus group interviews or the diaries. Categories and themes were discussed until agreement was reached.

The design sessions were video-recorded. Data from the supplementary field notes were transcribed by the first author. To become acquainted with the data, the video recordings were looked through numerous times while taking notes. The process was supported by involving a member of the researcher team (KK) who had not participated in the design sessions.

**Table 2:** Examples from the content analysis process; condensed meaning units, categories and subthemes corresponding to the three themes

Meaning unit (examples of each theme)	Category	Subtheme	Theme
“*Heart failure, that means that the heart has failed… it is not working any more… to me reduced pump function means that it is not pumping as it should… it is two completely different things… heart failure… it sounds awfully severe*” (Male 7, focus group)	Thoughts that concern the heart condition	Confusion about what heart failure is	Anxiety and uncertainty as driving forces for learning
“*At home… really feeling unwell and dizzy if I just make a few quick movements… and in such a way that it comes just by doing normal house chores – I am in doubt about if I should ignore it or? I took my blood pressure, 119/68 and later 130/90... I doubt if I can go for physical exercises... I have experienced these days before and I think if it is the “pressure”; we had a death in the family and I was “on”, had a lot of tasks… I think maybe that there is a tendency to dizziness when I have too much to do… not just physically, but when I’m “on”…*” (Female 4, diary)	Experiencing (unrecognizable) bodily sensations, unsure about what it is and how to respond	Trying to understand and make sense of bodily sensations/experiences	Managing my condition
“*I can’t, for instance, vacuum (clean) as I could before…now I have to plan much more…when I wash the floor I need to lie down for half an hour and then go on with the next floor…there are so many limitations…and I have been used to jumping around dealing with everything…so I find it hard … and when you are going out…you need to go somewhere where you can rest…I think that is probably the worst…*” (Female 2, focus group)	Domestic chores have become difficult/ challenging and require planning. The need to rest has become a new basic term / (condition of life)	Heart failure puts limitations on what you can do – on daily life. New ways to manage must be learned	Managing my daily life

This researcher conducted one- third of the analysis of the video material. A single video recording was considered to be a unit of analysis. Meaning units were recognized, condensed, and abstracted into categories in relation to the aim of the study at the manifest level in order to resolve.

“What was said” (Table 3). Categories were discussed until agreement was achieved. Repeated checks for accuracy as compared with the original audio and video recordings and diary text were done throughout the analyses of Steps 1 and 2.

**Table 3:** Examples from the content analysis; condensed meaning units, codes, subcategories and categories

Meaning unit (examples of each category)	Codes	Subcategory	Category
“*It needs to be a language where normal patients can keep up*” (Male 14, design session, website prototype)	Recognizable language Usual terms/daily expressions	Clear language and text	Adapting the content to the patient group
*“…you could also go in and write what you have experienced… it could be like a space for patients’ opinions or experiences …where you go in and say this and that... and say that was a good way… for those who are interested…”* (Male 13, design session, paper prototype)	Sharing experiences Expressing opinions Support to others	Peer support	Opportunities to interact with peers and professionals for support
*“…the website should rather have few things… and then you can “dig” into… the more topics the more difficult it gets to go through it… and some will simply skip it…”* (Male 15, design session website prototype)	Different topics Layers of information Accessibility and usefulness	Basic issues	Interface for learning

## Findings

### Learning needs experienced by patients between follow-up visits

The analysis of the focus group interviews and the patient diaries exposed three themes. Anxiety was a recurrent and predominant factor in all three themes and seemed to be the driver behind patients’ need for knowledge due to their questions and concerns. The first theme, “Anxiety and uncertainty as driving forces for learning”, was recognized as a learning-related issue permeating the patients’ daily lives. The themes, “Managing my condition” and “Managing my daily life”, elucidated how the patients’ learning needs arose from questions, challenges, and concerns triggered by daily situations and contexts. An example of meaning units and subthemes underpinning these themes is shown in Table 2. Below, the themes are described and supported by quotations.

### Anxiety and uncertainty as driving forces for learning

This theme highlights how feeling anxious in a new life situation with heart failure triggers questions and the need in patients to understand what the condition means to them.

Regardless of gender, the patients expressed strong emotions related to the concept of “heart failure” and being diagnosed with the condition. This seemed to affect their ability to understand the condition itself and it raised many thoughts and questions.

“Heart failure means that the heart has failed…it is not working anymore…to me, reduced pump function means that it is not pumping as it should… it is two completely different things… heart failure… it sounds awfullysevere.”(Male 7, focus group)

Topics such as the significance of the diagnosis and prospects were highlighted as important issues to know more about and caused great concern and anxiety for many patients. Some cited a lack of understanding of their own situation and expressed a need for more information about chances for a longer life, while others expressed doubt about whether or not the condition could be cured. These questions and anxieties occurred in everyday conversations with family members and seemed to affect and challenge the patients.

“Heart failure, can you die from that?...or what do you need to pay attention to?” (Male 12, diary)

The thought of having to experience several serious side effects caused anxiety and some saw this as unbearable and thus the prospect of a longer life was not appealing:

“They tell me that these beta-blockers… which I might have to take some day, will be able to prolong by life… but if my life is going to be like this… my ears are buzzing all the time… having problems with bursting into tears sometimes… all the time… then I would rather die a couple of years earlier… I really don’t want… it is unbearable” (Male 7, focus group)

The psychological aspect of heart failure was mentioned as the worst thing some patients had experienced. Feelings spanned from mood swings to a fear of death, and they wondered why emotions like these would come in connection with heart failure. In this context, they expressed a need for more information.

“It is probably the worst thing I have ever experienced… the psychological thing… it is like having a death sentence hanging over your head…” (Female 2, focus group)

### Managing my condition

Patients identified several areas where they wished to have more information in order to manage their condition. In particular, medication was a subject that preoccupied them.

The importance of the opportunity to obtain more information about the effects and impact of medication on the body and heart, reasons for increasing doses, the number of medications needed, side effects, and how to respond to all of this was stressed as being important by the patients.

“I think the drugs for the blood pressure… you get too little information… what effects they have.” (Male 7, focus group)

“I want more information about… why you need to increase the dose… when it is going so well – taking the other dose.” (Male 12, diary)

All patients experienced side effects and reflected on the need to learn more about these effects to enable them to manage their daily lives. They wanted, in particular, to know how they should respond to the side effects. Side effects were reported by several patients to affect them socially and mentally. The diaries revealed frequent experiences of side effects including, e.g., dizziness.

“I go around feeling dizzy all the time, as if I had been drinking... everything is buzzing… and when I walk on the street people think I am drunk… it is very uncomfortable.” (Female 2, focus group)

Despite being preoccupied with such strong emotions as anxiety, the patients had suggestions for their own learning.

 “… it could be interesting to see [ultrasonography] when you have… started the medication for blood pressure or the other blockers or whatever they are called… that the heart has started working again… and then you should be over it.” (Male 7, focus group)

Recognizing and reacting to bodily sensations experienced in daily life and contexts during activities was difficult for most patients. When these sensations occurred they wondered what they were and whether or not they could be ignored. They also wondered whether or not they could be critical and the consequences of overlooking them. Learning about sensations and knowing how to deal with them seemed to be necessary to manage daily life.

“At rehabilitation… I sat on the bike and was really out of breath... and I said (to myself) am I out of breath or do I have breathlessness? Is it dangerous or not dangerous?” (Female 1, focus group)

The possibility of follow-up visits was another important issue brought up by the patients. They considered these visits to be a “lifeline,” but lacked knowledge about what would happen when the visits ended.

“Now when I am finished with the medication part with the nurse… is there then no more follow-up… are there no more follow-ups?” (Male 8, focus group)

Having the opportunity to communicate with fellow patients and getting advice from other patients or healthcare professionals were expressed as important issues by patients. These needs were mostly triggered by specific situations or occurred in actual contexts, and the patients used such expressions as “being in the same boat” when they referred to other patients with the same condition and viewed this opportunity as a way to get support. Interactive elements for online support were also suggested.

“I need a website where I can search for different symptoms and someone to talk to.” (Male 12, diary)

### Managing my daily life

How to manage daily life surfaced as a third central theme. It covered how the condition challenged and impacted different everyday life situations. This resulted in a request to gain knowledge and skills concerning how to respond.

The work situation was an area where some patients experienced a change because of their heart failure condition. They described how they felt unsure about their future working situation and expressed a need to get information about who to turn to for help. Some described how specific work situations had made them question their ability to manage their current job tasks and had led to changed roles and identities. Consequently, questions about whether it was necessary to find a new job were expressed.

“I really got reminded of my limitations today at the staff meeting where we discussed 12-hour shift; the majority wanted them. I feel “ruled out” because I do not have energy for that and definitely not for three days in a row. I feel sad – should I look for a new job?” (Female 3, diary)

Limitations in daily activities and domestic duties leading to changes were other areas that patients experienced as challenging. Being in specific daily situations made them bring up the question of “how much”. They expressed uncertainty about what activities were “allowed” and found it difficult to get a precise answer to what would be the upper limit for participation in physical activity. The reason for and impact of physical exercise on heart failure was not clear to all patients.

 “I think I get different answers to how much I can pressure myself when exercising… the physiotherapist “dares more” when it comes to increasing the strain.” (Male 9, diary)

Most patients linked fatigue with taking medication; none linked it to the condition itself. Fatigue was a frequently experienced symptom in connection with a specific situation, and it created uncertainty. For some patients, fatigue resulted in a feeling of being “handicapped” and affecting what they could achieve on a daily basis.

“I also think it [fatigue] has to do with the medication you get… that it affects the body…” (Male 3, focus group)

### Patients taking part as co-designers of a website

Initially, two scaffolds for the co-design process were provided: (1) a low fidelity paper prototype of the website and (2) fifteen post-it topics featuring topics and subtopics in simple text descriptions. Topics and subtopics were based on the patients’ learning needs, which evolved from content analyses of focus group interviews and diaries. Post-it topics included:

   •   The condition (topic) - What is heart failure. Subtopics included, for instance: Life expectancy, causes for heart failure, psychological aspects.

   •   Medication (topic) - Patients with heart failure may need multiple types of medication. Subtopics included, for instance: Impact of medications on body and heart, reasons for increasing dose, the number of medications needed, side effects, and how to handle them.

   •   Daily life (topic) - What is a life with heart failure. Subtopics included, for instance: Work situation, restrictions on daily life and domestic duties, reasons for fatigue and how to deal with fatigue, and exercise.

   •   In addition, a post-it text included: Video/ultrasonography to illustrate the condition.

The post-it topics were used to outline the paper prototype of the website and were introduced to participants before use. They were not presented as final ideas, but were used to inspire ideas about the design of the website. Following the content analysis of the design sessions with the paper prototype, we revised and re-interpreted the patients’ ideas and built a prototype of the website in a free website environment. This revision informed decisions about how to focus the design and required that parts of the content were adapted to fit in a website context properly (e.g., shorter headings to fit an icon, etc.). The website included large icon images displaying such main topics as “the condition”, “daily life”, and a pull-down menu of the subtopics.

### Findings from the design session

The design sessions yielded the following three main categories: “Adapting the content to the patient group”, “Opportunities to interact with peers and professionals for support”, and “Interface for learning”. These categories represented the topics and elements reported as important by patients in the design of an educational website; each category had some subcategories. An example of meaning units and subcategories underpinning these categories are shown in Table 3.

### Adapting the content to the patient group

This category concerns how patients wished to access the educational website and the ways in which the content should be provided.

#### Getting in touch with the audience

Patients emphasized that the website should be easy to access and target the audience and their level of experience with and competence in the use of computers:

“...you need to consider that many of us are elderly…” (Male 13, design session, paper prototype).

Ideas about how this could be addressed by the design were put forward, e.g., by targeting the text and language of the site toward the target audience:

“...there is something about arranging the language to fit the age group.” (Male 14, design session, website prototype)

#### Clear language and text

A straightforward and understandable language without use of medical terms was viewed as essential and a key issue by the majority of the patients. One patient made the point that,

“it must be words that you can recognize… a broken arm is a broken arm... and not something with a fracture” (Male 15, design session, website prototype)

Some patients emphasized that failing to take this into account could result in patients deselecting important information, something the patients could be reluctant to be open about due to the embarrassment of not understanding. They deemed it crucial that the text and headings should be kept short and clear and easy to read. One patient further linked on to how acute illness or chronic diseases change one’s ability to comprehend things:

“Especially when admitted, some patients become very worried about whether they are going to die tomorrow. … your ability to understand goes down to a level which is not normal or usual because you have these dark thoughts...” (Male 14, design session, website prototype)

For some patients, it was important that the text should be carefully written and not be “frightening” since otherwise it could be overwhelming and scary to read. Some thought other patients preferred fewer details. They expressed the opinion that videos/demonstrations should be simple, without blood, and were concerned that scenes from, e.g., an operating room could be overwhelming. One stated:

“Yes, but I think you should be a bit careful… I had a test of my coronary arteries and, well, that gives a bit of blood… maybe you could do that without so much blood.” (Female 5, design session, paper prototype)

It was also suggested that video material and illustrations could be a way to prepare patients for a test and, likewise, patients who had already undergone tests could share their experiences online.

#### Relevant content

The topics were recognized by the patients in the design session and they also advocated additional content for the website. One found it essential to include a description of the continuity of care for patients with heart failure:

“What the care is like… and what the treatment and follow-up are like… what options there are.” (Male 13, design session, paper prototype)

Others suggested acquiring knowledge about the reasons for treatments and what to expect, and they stressed the need for more detailed information about the effects of medication on vital organs. They also frequently pointed out that the focus of the topics addressed should be on relevant knowledge usable in everyday life situations, such as how to respond to side effects. One patient said:

“It is essential to know how you should deal with an upset stomach due to medication.” (Male 14, design session, website prototype)

#### Multimedia applications – to support learning

Video materials showing different tests or treatments within the continuity of care, together with written text, were suggested. Providing information in two ways to enhance understanding was emphasized by some patients. Suggestions for how this could be done, e.g., by ultrasonography to teach about the effects of medication on the heart since patients often did not feel the effects were put forth in Step one. However, some patients had contradictory views on this issue.

### Opportunities to interact with peers and professionals for support

This category deals with issues concerning support and interaction through the website.

#### Peer support

The website should provide ways to identify with others and to hear from those who have already been through treatments and tests. The patients felt that there was a gap in information and knowledge with respect to, e.g., tests and medication which the website could help to bridge. The experiences of others living with heart failure or having gone through tests or treatments were considered to be of central importance for providing support. The patients suggested free text space to write about their own experiences and discussion forums and saw especially the latter as a good opportunity to receive advice from others and a framework for sharing experiences. Perspectives differed on this issue since some stressed how this could lead to situations where patients would write about their health getting worse and possibly affect those attending the forum and making them feel obliged to act.

#### Ways and means to receive support from professionals

By providing a frequently asked question section, the patients felt they could learn from the queries of others. The question of enabling users to write questions to professionals at the clinic in a frequently asked question section was also raised. This was viewed as a positive option although it was mentioned that answers should be provided within a reasonable period. Chats or online consultations with professionals were viewed as important parts of the website by some patients and were argued to be likely to save time and be more flexible in relation to the patients’ everyday life. The patients emphasized that the intention behind these options should be clear from the beginning.

### Interface for learning

This category concerned the design of the interface of the website.

#### User preferences

Patients suggested using the expression “Questions and answers” instead of abbreviations. Lengths of texts and headings were considered important since a short text was seen as a way to keep focus on the subject.

#### Basic issues

The structure of the website was regarded as important and it was suggested that content concerning, e.g., medication should only cover the most general information. However, the patients also stressed the need to have access to specific advice on how to respond to side effects. The patients also expressed other seemingly inconsistent views. Some felt they were very well informed about their condition. Nonetheless, they called for more explanations concerning medication and reasons for treatment options, which they thought the website should include as well.

Patients suggested toolbars provide layers of information, and they thought that it was feasible for this target group to use toolbars to find information. There were disagreements on the number of topics (illustrated as an icon and serving as links) displayed on the front page. It was suggested to cut the number of topics down from 6 to 2–4 to make it easier to get an overview. However, later, more topics were suggested to embrace more subjects and situations.

Most patients thought it was essential to keep menu headings and texts short. There was some disagreement on how much a topic should embrace and also about understanding the language used for describing the topics. It was considered necessary to balance the description of the content in an accurate and exciting way, avoiding that it was either too specific or too general.

The patients wanted a clear and concise front page. One said:

“I think you should make it simple.” (Female 5, design session, paper prototype).

Later, however, the same patient wished to have more options. Pictures, illustrations, contact information, video material, chats, “Questions and answers,” online consultation, a discussion forum, forum space to share experiences, written text, links, and new knowledge (evidence) were all elements regarded as essential by the patients for the website and : as important means to establish support and obtain an agreeable atmosphere.

## Discussion

A significant finding in this study is the anxiety patients experience when confronted with heart failure. One side of

this coin is that the anxiety becomes a driving force to learn how to manage daily life. Since it is very important for patients to understand the condition, they are highly motivated to learn. This is in line with Illeris’^42^ and Kneck and colleagues’ descriptions^43^ of how emotions are part of every learning process and can serve as a driver for learning. Emotions were most dominant in the diaries, which revealed routines and activities in the patients’ daily lives that were highly influenced by these emotions. The other side of the coin was that, due to their anxiety, many patients seemed to be affected in their ability to engage in new learning, which hampered their understanding of the condition itself. In the present study, we saw that patients had difficulties in understanding the language used by healthcare professionals, which could explain why they do not acquire this understanding. This could intensify the patients’ anxiety and uncertainty even more.

The patients in this study identified several areas in which they needed more knowledge and skills to understand and manage the condition effectively in their daily lives. In general, our findings concerning patients’ learning needs are in line with prior work.^15^^,^^19^^,^^22^^-^^25^ These studies have reported learning needs to involve acquiring information about, e.g., medication, signs and symptoms, risk factors, and prognoses. Our findings also highlight other aspects of these areas which the patients found to be important to learn about and understand in order to manage their daily lives. What is unique in our findings is that they showed how patients’ learning needs were often influenced, connected, and triggered by everyday situations and contexts, placing a greater emphasis on the importance of healthcare professionals being aware of the patient's life and everyday activities. We found, moreover, that most questions arise at home, which stresses the need to take the patients’ daily life situations as the starting point for learning.

We stress the patients’ perspective, and they were able to identify several learning needs such as, for instance, need to learn about bodily sensations in the context of their condition. In this regard, they frequently had problems with understanding such sensations in connection with everyday situations, which made it difficult for them to respond adequately. The need to develop the ability to interpret signs and symptoms and respond to them corroborates findings in other studies.^44^ Our findings also point out other areas where the patients had a poor understanding and wished to gain more knowledge, including the psychological impact of heart failure, challenges in daily life (e.g., fatigue), work situations, the extent and reasons for heart failure medication, what would happen when their follow-up visits end, and where they would be able to get support and help.

It has been suggested that involving users contributes to making websites more meaningful.^45^ Our findings showed that patients were, in general, open to the co-design process and the tools, serving as “scaffolds”. This allowed them to envision the future website, express their thoughts and ideas and thus help to point out how to focus and address topics to make the content meaningful for future users. This process served the purpose of discovering elements for additional inquiry and modification.

While diverse kinds of structures for web-based patient education have been put forward,^46^^,^^47^ our findings disclosed which elements and structure the patients requested and thought would be relevant and useful in managing everyday situations, including information supporting their own ability to respond. These inputs and ideas can be important for a website attempting to support patients’ learning about heart failure.  In our study, the patients were able to point out elements concerning both details of the conceptual design, e.g., what the website should look like and the physical design regarding, e.g., the atmosphere on the interface, images, menus, etc. They believed that the website should be easy to access and target the audience with regard to form and content, as well as being written in an understandable language without medical jargon. This has been reported previously.^15^ More importantly, the information should be easy to access and the users should not have to struggle with technology at the expense of getting understandable information about the topic at hand.^45^ The findings also indicated that the patients wanted such interactive elements to be part of the website as discussion forums with other patients, chats with professionals, online consultations, and “Question and Answer” facilities. The positive effect on the quality of life and decreased use of healthcare services has been reported^48^ when a website with health information included a discussion group. Patients in the present study argued about how a discussion forum could decrease anxiety and stress. In our study, we saw that patients suggested additional topics, as well as different multimedia applications, such as videos, to prepare for tests and as a way to support learning. Proposing ideas to meet the requirements of a website is central in the design process,^49^ and video has been described as a way to support retention by means of illustrations and other tangible impacts.^45^ The patients in this study expressed conflicting views concerning the details of these videos, thereby illustrating that patients do not always know what they want or need.

Although patients contributed valuable knowledge and ideas, our findings revealed more conflicting suggestions for the website. In general, the patients expressed a wish for brief information concerning, for instance, medication, but stressed the importance of having access to additional specific advice on how to deal with, for instance, side effects and voiced a wide range of issues concerning which medication the website should include. Opposing views were similarly expressed concerning the number of topics included based on an understanding of learning needs. They expressed an appreciation of certain topics; however, they wanted few topics, but, on the other hand, requested a website that allowed the users to search for a wide spectrum of information. This seems to suggest a design which should both meet the need of a “patient in need” and be a place for learning for users requesting in-depth knowledge.

### Limitations of the study

One limitation is that this research is based on experiences from a single institution. Four participants declined to participate. The reasons were either that they did not feel up to it or felt unsure about whether they could contribute anything useful because they believed that they had limited technical skills. Their perspectives could perhaps have provided important information since many other patients with this condition might feel the same and supporting the learning needs of this patient group might require other strategies when designing online learning tools. Two (out of the four) participants forgot to turn up for the design sessions and they could have perhaps provided new ideas to push the process even further. It could be seen as a limitation that only four patients participated in the design session; however, the goal was to involve patients, listen to their ideas, support their feeling of ownership and learn from the website in order to get an idea of “how to get there” in terms of supporting patients’ learning needs, using an educational website. The use of mixed methods allowed us to get the findings from the first step validated in the second step. By providing a rich description of the context of this research including heart failure patients varying in age and by describing the theoretical foundation for the research and presenting our findings with illustrative quotations, we believe others may reflect our findings into other contexts. Our analysis allowed for some insight into the characteristics of patient learning and findings that can hopefully be transferable to patient learning in other diseases.

## Conclusions

In this study, the learning needs of patients with heart failure between their follow-up visits at an outpatient clinic were uncovered from the patients’ perspective. We argue that patients’ learning needs are many-faceted and influenced by daily situations and emotions. They comprise an understanding of the condition, its course and treatment, its life-changing impact on daily life, stressing a need to know how to respond, and understanding who to turn to for help and support in the future. Anxiety and uncertainty drove the learning needs discovered here and they were triggered by everyday situations and contexts, thus emphasizing the importance of gaining knowledge that focuses on everyday life and on how to respond to side effects, bodily sensations, etc. The lack of knowledge, questions, and concerns when facing daily activities become the point of departure for patients’ learning needs and healthcare professionals need to be aware of this in encounters with patients where the aim is to support their self-care.

We argue that patients’ learning needs may be supported by a website and that involving patients as co-designers in the design process can help to point out how to focus and address the topics in order to make the content meaningful for the patients and managing daily situations. Our findings show the need for the educational website to be adapted to the patients’ physical/cognitive/emotional state and everyday life situation. Patients may, however, not always know what they want and need and, therefore, many design decisions will still be left with the designers. Our study highlights the fact that patients’ learning needs can help drive the content of an educational website and that it should target the audience, be clear, well-structured and relevant, present an agreeable atmosphere, use understandable terms and language, and have interactive elements, with peer and professional support, as well as offer facilities for collaboration. This form and structure can help to support patients’ learning needs between their follow-up visits.

### Implications for medical education and recommendations for future research

In this study, patients are regarded as learners rather than passive recipients of information about their illness and care. Involving learners in the discovery of their own learning needs has been emphasized previously^9^ and this study showed what help patients need to understand their illness and how to manage their self-care from their own perspective. We also found how patients struggle with understanding the language used by professionals, as well as with feelings of anxiety which impeded their learning process. This knowledge could prove to be useful for the development of new patient learning activities within the area of heart failure or heart disease in general. Healthcare staff need to understand the importance of this matter and learn to put greater emphasis on giving patients the opportunity to describe their own learning needs or gaps in knowledge between where they are now and where they want to be. Healthcare staff should also encourage patients to bring forth ideas to meet these needs and how their anxiety can be eased since this could support them in becoming independent and self-directed learners. Moreover, the role and impact of anxiety and uncertainty need to be understood and taken into consideration when designing patient learning in general. Since patients struggle with understanding the language used by healthcare professionals, the professionals need to express themselves in a more simple language. The patients in this study came up with many useful ideas and focused to a great extent on the development of interactivity of the website, which may enhance the learning experience. Patients can act as co-designers and should be invited to take part in the design process since they, in this role, can help point out how topics can be addressed and how they could be useful and meaningful for managing daily situations and relevant problems, which is of interest to the adult patient learner.^9^^,^^42^

There is a need for further studies on the learning needs of patients with heart failure from the patient’s perspective in order to design the most effective educational program for patient learning. Future research needs to investigate how we can design a supportive learning environment for the patients and how to manage the sometimes contrasting and conflicting opinions voiced by different patients. Another important issue is to consider how to make this knowledge a part of undergraduate and continuous medical and healthcare education.

### Acknowledgments

We are very grateful to the patients who participated and shared their experiences with us. We also wish to thank the Department of Cardiology at Aarhus University Hospital for supporting this study.

### Conflict of Interest

The authors declare that they have no conflict of interest.
